# Evaluation of oral brush liquid-based cytology for oral squamous cell carcinoma: a comparative study of cytological and histological diagnoses at a single center

**DOI:** 10.1186/s12903-023-02839-w

**Published:** 2023-03-11

**Authors:** Katsutoshi Kokubun, Kei Nakajima, Kei Yamamoto, Yoshihiko Akashi, Kenichi Matsuzaka

**Affiliations:** Department of Pathology, Tokyo Dental College, 2-9-18 Kandamisaki-cho, Chiyoda-ku, Tokyo, 101-0061 Japan

**Keywords:** Cytology, Pathology, Liquid-based cytology, Diagnosis, Oral cancer

## Abstract

**Purpose:**

Liquid-based cytology is highly useful in oral cytology. However, there are only few reports on the accuracy of this method. The current study aimed to compare oral liquid-based cytological and histological diagnoses and to evaluate items that should be considered in oral cytological diagnosis for oral squamous cell carcinoma.

**Methods:**

We included 653 patients who underwent both oral cytological and histological examinations. Data on sex, specimen collection region, cytological and histological diagnoses, and histological images were reviewed.

**Results:**

The overall male-to-female ratio was 1:1.18. The tongue was the most common specimen collection region, followed by the gingiva and buccal mucosa. The most common cytological examination result was negative (66.8%), followed by doubtful (22.7%) and positive (10.3%). The sensitivity, specificity, positive predictive value, and negative predictive value of cytological diagnosis were 69%, 75%, 38%, and 92%, respectively. Approximately 8.3% of patients with a negative cytological diagnosis had a histological diagnosis of oral squamous cell carcinoma. Furthermore, 86.1% of histopathologic images of cytology-negative squamous cell carcinomas exhibited well-differentiated keratinocytes lacking atypia on the surface. The remaining patients developed recurrence, or they had low cell counts.

**Conclusion:**

Liquid-based cytology is useful in screening oral cancer. However, a cytological diagnosis of superficial-differentiated oral squamous cell carcinoma is occasionally inconsistent with the histological diagnosis. Therefore, histological and cytological examinations should be performed if tumor-like lesions are suspected clinically.

## Introduction

Oral cancer is the 16th most common neoplasm worldwide, with an estimated 355,000 new diagnoses and > 177,000 deaths in 2018 [1]. The most common sites of oral cancer are the tongue, gingiva, and floor of the mouth, accounting for more than half of all the cases. Oral cancer arises primarily from epithelial cells, and almost 90% of cases originate from squamous cell carcinoma (SCC) [2]. Surgical resection is usually the treatment of choice for oral cancer [2]. Overall survival rates vary by region, geography, and stage of the disease. In Japan, for example, approximately 60% patients survive 5 years after diagnosis [3]. Regarding postoperative satisfaction, appearance, swallowing dysfunction, dry mouth, and oral functional deficits are significantly better in patients with early-stage versus advanced-stage cancer [4]. Therefore, early diagnosis is important in preserving quality of life (QOL). However, mortality is still an issue.

Accurate and early diagnosis of oral cancer and mucosal diseases is essential for developing treatment strategies and improving patient prognoses [5]. Histological examination is widely used as a general definitive diagnostic method [6]. This method is as invasive as a blood test, which can be burdensome for patients. However, a histological examination is necessary for obtaining a definitive diagnosis. Oral cytology is less invasive and easier to perform than histological examination and can be a useful screening method for oral mucosal diseases [7].

Exfoliative cytology using an oral brush can help dentists and other physicians determine whether oral lesions are malignant or in an early, curable stage [8, 9]. Exfoliative cytology is a simple, safe, and reliable method of microscopically examining cells that have been shed or desquamated from the mucosa. Exfoliative cytology includes the conventional method and liquid-based cytology (LBC) [10]. The conventional method involves chairside scraping of the oral mucosa and directly smearing it on a glass slide. This method requires proper technique because mishandling can alter the morphology of the collected cells. Conversely, in LBC, cells are spread in a fixative solution to create a thin layer of cells on the slide. Therefore, LBC, which is widely used, does not require complicated manipulations. In addition, the cell collection volume is larger and specimen artifacts produced by bleeding and saliva are reduced in LBC compared with the conventional method [7, 11]. However, fewer studies have focused on the accuracy of LBC than the number of studies focused on the conventional method [7]. Furthermore, the diagnostic accuracy of LBC over histological diagnosis has not been studied in detail. Therefore, the purpose of this study was to examine whether LBC is sufficient as a standard method for screening suspicious lesions in the oral cavity and to understand the limitations of LBC. For this, we retrospectively compared LBC results with histological diagnoses.

## Materials and methods

### Patient characteristics

Patients with clearly visible oral lesions, which showed apparent variation from the normal healthy mucosa, presenting to the Tokyo Dental College Hospital for new or follow-up appointments were screened for the study. Patients with an oral mucosa abnormality that appeared clinically benign (minimally suspicious) and did not have an obvious etiology, such as trauma, were included in the study.

### Data collection

We included 653 patients who underwent oral cytology and histological examination at our hospital from January 2018 to December 2020. The mean age of men (n = 299) was 58.6 years, and the mean age of women (n = 354) was 60.4 years. The mean age of all the patients was 59.6 years ± 16.4 (standard deviation, SD). The clinical diagnoses included 330 malignancies, 131 cases of leukoplakia, 76 cases of lichen planus, 51 cases of benign tumors, 22 cases of papilloma, 22 cases of inflammation, 12 cases of oral epithelial dysplasia, 7 cases of erythroplakia, and 2 cases of pemphigus. Patient characteristics, including age, sex, specimen collection site, and cytological and histological diagnoses, were examined. The specimen collection sites were the tongue, gingiva, floor of the mouth, buccal mucosa, palatal plate, and lips.

### Laboratory procedures

Cytology was performed using BD SurePath™ (Becton, Dickinson and Company, Tokyo, JAPAN), and cell samples were collected using the Orcellex® Brush (Becton, Dickinson and Company, Tokyo, JAPAN). After twisting the brush ten times, the head of the cell collector was transferred directly into an alcohol-based liquid in the BD SurePath™ Collection Vial (Becton, Dickinson and Company, Tokyo, JAPAN) and transported to the laboratory. The cells were stained with Papanicolaou stain according to the standard procedure. Oral pathology diagnostic experts confirmed that the samples were appropriate for cytological diagnoses. A detailed cytological assessment of cell quality and yield was performed using the modified 2014 Bethesda Cervical Cytology grading system [12]. The assessment included cellularity, quality of preparation, cell types, microbiota, presence of leucocytes/inflammatory cells, presence of artifacts, and, when applicable, dyskaryotic epithelial changes or features suggestive of SCC. Slides were reported as inadequate if they exhibited poor cellularity, poor fixation (air-dried), and/or thickly spread or obscured elements.

### Cytological diagnosis

**Fig. 1:**
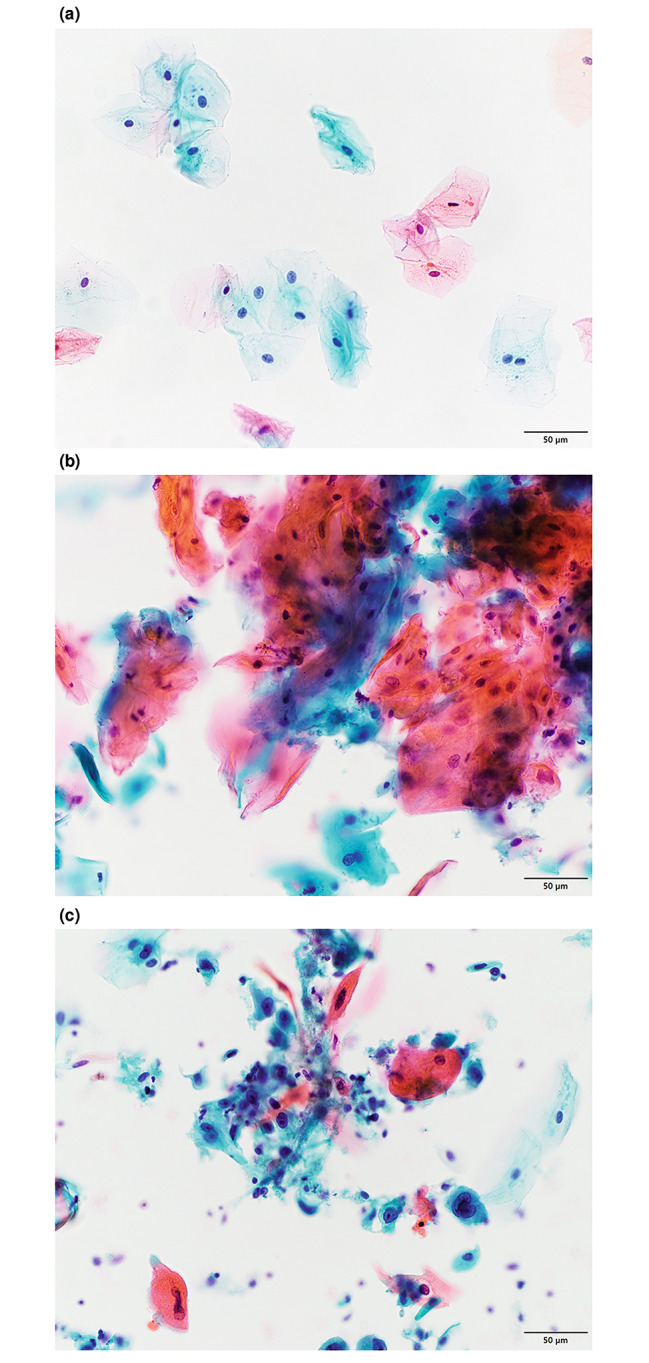
(a) Negative for tumor cells. Normal superficial and intermediate squamous cells. The background is completely clear. All nuclei are round to ovoid with smooth contours and finely granular, evenly dispersed chromatin. (b) Doubtful for tumor cells. Sheets of atypical squamous cells. The nuclei are haphazardly oriented, and the axes of different nuclei are not parallel. Orangeophilic cells represent abnormal variations in nuclear size and hyperchromasia. (c) Positive for tumor cells. A loosely cohesive sheet of highly atypical, immature basal, or para-basal-like squamous cells. The nuclear-to-cytoplasmic ratio is markedly increased. Nuclei are highly hyperchromatic and chromatin is coarse. Some cells show an irregular nuclear contour and/or small nucleoli. Some bizarre orangeophilic cells represent atypical keratinization. Those cells may have opaque, nearly black nuclei with smudged chromatin

All Papanicolaou-stained LBC slides were independently assessed by at least two of five oral pathology diagnostic experts. Any discrepancy in the assessment was settled by consensus. All participating experts had passed the board examination of the Japanese Society of Pathology for oral pathology and had been practicing for > 7 years. The cytological diagnosis was divided into three levels: negative (Fig. 1a), doubtful (Fig. 1b), and positive (Fig. 1c). A “positive” diagnosis signified that malignant cells were observed; “doubtful” signified malignant cells were suspected but not confirmed; and “negative” signified no atypical cells were observed in the smear [13].

### Histological diagnosis

**Fig. 2:**
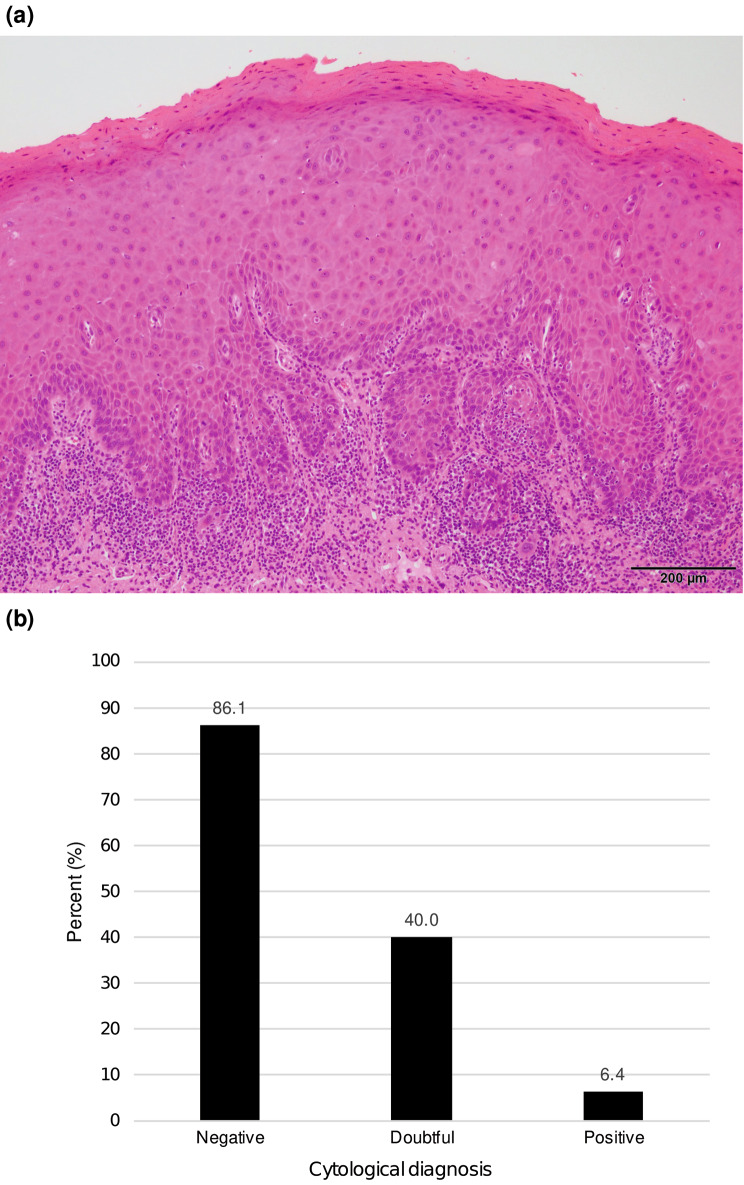
(a) Typical superficial-differentiated squamous cell carcinoma in a case with false-negative cytological diagnosis, with the deep epithelium showing downward proliferation and tumor cell invasion in the underlying tissues, and keratinocytes in the superficial layers of the epithelium without atypia (b) Percentage of superficial-differentiated squamous cell carcinoma in each cytological diagnostic category. The percentages are shown above the columns

Histological slides were stained with hematoxylin-eosin and examined by at least two oral pathology diagnostic experts. Another oral pathology diagnostic expert was consulted to obtain a diagnosis by consensus in case of discrepancies. The histological findings were classified into nine categories: squamous cell carcinoma, high-grade dysplasia, low-grade dysplasia, epithelial hyperplasia, lichen planus, inflammation, benign tumor, other malignancy, and others. “Superficial-differentiated SCC” was defined as a type of squamous cell carcinoma accompanied by well-differentiated keratinocytes lacking atypia on the surface (Fig. 2a). The histology of all SCC cases was reviewed to determine the percentage of superficial-differentiated SCC. The evaluation was performed independently by four oral pathology diagnostic experts, who determined the outcome of each case by majority vote.

### Statistical analysis

Data were entered into a database using Microsoft Excel (Microsoft Inc., Redmond, WA, USA). To examine diagnostic ability via cytological and histological examinations, histology was classified as negative and positive (SCC) and cytology as negative (negative) and positive (doubtful, positive). Then, sensitivity, specificity, accuracy, positive predictive values, and negative predictive values were calculated. All statistical analyses were performed using Prism version 9 (GraphPad Software, CA, USA).

## Results

The participants were comprised of 54% women (n = 354) and 46% men (n = 299) (Table 1). The cytological results were classified as negative in 436 (66.8%) patients, doubtful in 148 (22.7%), positive in 67 (10.3%), and inadequate material in 2 (0.3%). The tongue (n = 259 [39.7%]) was the most common collection site, followed by the gingiva (n = 247 [37.8%]), buccal mucosa (n = 93 [14.2%]), palate (n = 40 [6.1%]), lip (n = 13 [2.0%]), and floor of the mouth (n = 1 [0.2%]) (Table 2).

**Table 1:** Number of patients

	Male patients	Female patients	Total number of patients
Negative	186	250	436
Doubtful	78	70	148
Positive	35	32	67
Inadequate material	0	2	2
Total	299	354	653

**Table 2:** Number of cytological diagnoses in terms of individual site

	Tongue	Gingiva	Buccal mucosa	Palate	Lip	Floor of the mouth	Total
Negative	156	179	66	29	6	0	436
Doubtful	60	49	23	10	5	1	148
Positive	42	18	4	1	2	0	67
Inadequate material	1	1	0	0	0	0	2
Total	259	247	93	40	13	1	653

In patients with a negative cytological diagnosis, several lesions had weak atypia, which was characterized by abnormalities such as epithelial hyperplasia, lichen planus, and inflammation (Table 3). Of 436 patients with a negative cytological diagnosis, 36 (8.3%) had a histological diagnosis of SCC. Among them, one had recurrence. Based on histological diagnosis, tumor cells were located only in the deep margin of the lesion. Of 67 patients with a positive cytological diagnosis, 47 (70.1%) and 13 (19.4%) had a histological diagnosis of SCC and oral epithelial dysplasia, respectively. In patients with a doubtful cytological diagnosis, the histological diagnoses included SCC, oral epithelial dysplasia, inflammation, epithelial hyperplasia, and oral lichen planus.

**Table 3:** Cytological versus histological diagnosis

	Negative		Doubtful		Positive		Inadequate material	Total
	Number	Percent	Number	Percent	Number	Percent	Number	
Squamous cell carcinoma	36	8.3	35	23.6	47	70.1	1	119
High-grade dysplasia	23	5.3	19	12.8	9	13.4	0	51
Low-grade dysplasia	35	8.0	19	12.8	4	6.0	0	58
Epithelial hyperplasia	80	18.3	15	10.1	1	1.5	0	96
Lichen planus	63	14.4	14	9.5	0	0.0	0	77
Inflammation	55	12.6	18	12.2	3	4.5	0	76
Benign tumor	42	9.6	10	6.8	0	0.0	0	52
Other malignancy	0	0.0	2	1.4	0	0.0	0	2
Others^1^	102	23.4	16	10.8	3	4.5	1	122
Total	436		148		67		2	653

^1^Others include no malignancy and epulis

The sensitivity of cytology for detecting cancer cells was 69%, and the specificity for detecting non-neoplastic cells was 75% (Table 4). Its positive and negative predictive values were 38% and 92%, respectively.

**Table 4:** 2 × 2 contingency table for the cytological diagnoses compared to histological diagnoses, the golden standard

Cytological diagnoses	Histological diagnoses	
	Positive (SCC)	Negative (Non-SCC)
Positive	82	133
Negative	36	400

Test	%	
Sensitivity	69	
Specificity	75	
Accuracy	74	
Positive predictive value	38	
Negative predictive value	92	

Based on a histopathologic evaluation of false-negative cytological and histological diagnosis of SCC, 31 (86.1%) of 36 patients had superficial-differentiated SCC (Fig. 2b). Among them, nine had verrucous SCC. Of the 35 patients with doubtful cytological and histological diagnoses of SCC, 14 (40.0%) presented with superficial-differentiated SCC and 1 with verrucous SCC. In contrast, only 3 (6.4%) of the 47 patients with positive cytological and histological diagnoses of SCC had superficial-differentiated SCC.

## Discussion

Oral cancer should be appropriately diagnosed to determine treatment course, and early diagnosis is beneficial. There are two types of diagnosis (oral cytological and histological). Oral cytology is a widely used simple, noninvasive test. In addition, methods for the early diagnosis of oral cancer, such as oral cancer screening, are actively promoted, and they are considered effective for the early diagnosis of oral cancer and the detection of recurrent tumors. However, oral cytological and histological diagnoses are not always consistent, and a definitive diagnosis cannot be obtained via oral cytology alone [14].

The male-to-female ratio of patients who underwent cytology at our hospital was 1:1.18, with a slight tendency toward female predominance. This sex difference was similar to that reported in other clinical studies on oral mucosal diseases [15]. In this study, the tongue was the most common specimen collection region, followed by the gingiva. Based on the 2018 data from the Japan Society for Head and Neck Cancer Registry Committee, the incidence rates of oral cancer in Japan according to region were 54.7% in the tongue, 23.3% in the gingiva, and 7.8% in the buccal mucosa [16]. Moreover, the results of these regions coincided with those of the specimen collection regions in the present study, indicating that specimens were collected from regions where SCC was most frequently observed.

Recently, LBC is widely used in oral cytology. LBC is also used for standardizing specimen preparation to control the accuracy of cervical screening and is useful in oral cytology [7, 14, 17–25]. More cells can be collected via LBC compared with conventional abrasion cytology [26]. The oral mucosa has more keratinizing lesions than the cervix; thus, the cell collection volume is low, which is disadvantageous. However, this issue can be addressed with LBC in the oral region. Several studies have assessed the accuracy of cytology in the oral cavity [7], but only a few reports focused on the accuracy of LBC, which has a sensitivity of 59.2–97.53% and a specificity of 50.6–99% [14, 18, 19, 21, 23, 25, 27, 28]. The sample sizes in these reports ranged from 89 to 1352. We believe that our study is significant because it examined a relatively large sample size compared with previous reports, and its accuracy is similar to that of other studies.

There were a certain number of false-positive and false-negative results in this research. That is, 36 patients had false-negative results (rate: 8.3%). In general, the cytological diagnosis of oral SCC involves the evaluation of superficial cells stained with orange G with Papanicolaou stain and medium or deep-layer cells stained with light green. It is necessary to evaluate the diversity of superficial cells, such as the appearance of the cytoplasm with a thick or bright stain and hyperchromatic nucleus. In the mid- and deep-layer cells, the nucleus-to-cytoplasmic ratio is high, and the appearance of atypical cells with abnormal nuclear shape and size should be evaluated. A positive diagnosis can be made if there are clearly atypical cells in the middle and deeper layers of the cell layer. However, in cases where only mild atypia of the superficial layer of the cell layer is detected, the diagnosis is less than sufficient. Oral squamous cells are highly differentiated compared with the uterine and esophageal squamous cells [29]. Therefore, in some cases of oral SCC, the superficial layers of the squamous epithelium are highly differentiated, even though the middle to deep layers of the squamous epithelium are highly atypical [25]. In SCC of the cervix, epithelial atypia often originates from the basal layer, and it is replaced in all layers before developing into the intraepithelial and then an invasive carcinoma [30]. Although total displacement carcinomas also exist in the oral squamous epithelium, they are far more likely to be superficial-differentiated than those of the cervix [30]. Therefore, if only the superficial layer is evaluated, they may be underdiagnosed.

The current study evaluated the histological diagnosis of patients with a negative cytological diagnosis. Results showed that these patients had a histological diagnosis of SCC. In total, 31 (86.1%) of the 36 patients presented with superficial-differentiated SCC; these were accompanied with well-differentiated keratinocytes lacking atypia on the surface (Fig. 2a). Among them, nine presented with verrucous SCC. In these cases, cytological diagnosis alone is challenging because only superficial cells can be obtained, and it is not possible to detect highly atypical cells. Therefore, it is difficult to collect tumor cells via oral cytology in superficial-differentiated SCC. In general, oral cancer is challenging to diagnose via oral cytology in borderline lesions because invasion is often present in the deeper layers of the lesion even if superficial atypia is weak [25]. Based on the current study, it is challenging to evaluate the malignant potential of superficial-differentiated SCC via oral cytology, and a histological diagnosis is necessary. The causes of false-negative results include poor specimens and cell determination errors [15]. Poor-quality specimens can be caused by low cell counts, overlapping cells, blood cell coating, and specimen dryness. Cell determination errors are caused by overlooking atypical cells caused by low atypical cell counts, thereby underestimating the degree of cellular atypia and recognizing cancer cells as non-epithelial cells. One patient with a negative cytological diagnosis presented with recurrent SCC. In this case, tumor cells were present only in the deep part of the lesion, and no malignant findings were observed in the coated epithelium. In addition, 4 of 36 patients had low cell counts, thereby making it difficult to detect atypical cells, which may have led to underestimation. False-negative cytology result is more likely to be obtained if the exposed cell area for diagnosis is extremely small or proliferation is significantly limited [31]. False-negative results are likely to occur because basal or parabasal-like atypical cells are challenging to correct, which prevents the collection of useful cells for cytological diagnosis. Sekine et al. [14] reported a false-negative rate of 22.2%, which was acceptable in oral cytology. In the study of Remmerbach, the false-negative rate decreased slightly when LBC was applied instead of the conventional method. However, there was still a significant number of false-negative results [21]. False-negative cytology results may aggravate untreated cancers, which cannot be further treated or followed-up. Therefore, oral cytology itself should be further improved before it can be considered a completely reliable method. Therefore, the rate of false-negative cytology results must be decreased.

In this study, some patients had false-positive results. In several cases, although there was a positive cytological diagnosis, the histological diagnosis was inflammation. Malignant tumors often present with several atypical cells with nuclei. However, these cells are often found in inflammatory conditions such as ulcer margins and Candida infections [32]. Patients with false-positive results presented with cells with large nuclei due to reactive changes caused by inflammation.

In relation to these reasons, false-negative and -positive cytological diagnoses may be obtained. Particular attention should be paid to superficial-differentiated SCC, recurrence, and inflammation, as cytology and histology reports may be dissociated. Cytology is a minimally invasive tool that can be used to obtain a definitive diagnosis. However, it cannot be used alone. Histological diagnosis should be comprehensively assessed based on clinical findings, including gross findings, and disease course. It is important to make a comprehensive diagnosis by considering clinical findings and, if necessary, histological diagnosis according to lesion condition.

The current study had several limitations. That is, it was a single center, cross-sectional research. Thus, there was risk of bias. However, only few reports have compared the cytological and histological diagnoses of LBC. Therefore, this study is significant because it included a relatively large number of cases.

In conclusion, this study retrospectively compared the consistency between oral cytological and histological diagnoses among patients at our institution for the last 3 years. Some patients with a negative cytological diagnosis had a histological diagnosis of SCC, oral epithelial dysplasia, epithelial hyperplasia, and inflammation. Approximately 8.3% of patients with a negative cytological diagnosis had a histological diagnosis of SCC. Therefore, histological diagnosis must be obtained even in patients with a negative cytological diagnosis, and appropriate treatment should be provided if tumor-like lesions are suspected clinically.

## Data Availability

The datasets generated and/or analyzed during the current study are available from the corresponding author on reasonable request.
